# A Longitudinal Follow-Up Study of Saffron Supplementation in Early Age-Related Macular Degeneration: Sustained Benefits to Central Retinal Function

**DOI:** 10.1155/2012/429124

**Published:** 2012-07-18

**Authors:** M. Piccardi, D. Marangoni, A. M. Minnella, M. C. Savastano, P. Valentini, L. Ambrosio, E. Capoluongo, R. Maccarone, S. Bisti, B. Falsini

**Affiliations:** ^1^Dipartimento di Scienze Otorinolaringoiatriche e Oftalmologiche, Universita' Cattolica del Sacro Cuore, 00168 Roma, Italy; ^2^Istituto di Biochimica Clinica, Universita' Cattolica del Sacro Cuore, 00168 Roma, Italy; ^3^Dipartimento di Scienze Oftalmologiche, Universita' degli Studi di Napoli Federico II, 580131 Napoli, Italy; ^4^Dipartimento di Scienze e Tecnologie Biomediche, Università degli Studi dell'Aquila, 67100 L' Aquila, Italy; ^5^Scienze Cliniche ed Applicate Biotecnologiche, DISCAB, Universita' degli Studi dell'Aquila, 67100 L' Aquila, Italy; ^6^ARC Centre of Excellence in Vision Science, The Australian National University Canberra, ACT 0200, Australia; ^7^Istituto Nazionale Biostrutture e Biosistemi, 00136 Roma, Italy; ^8^Istituto di Oftalmologia, Universita' Cattolica del Sacro Cuore, Lgo F. Vito 1, 00168 Roma, Italy

## Abstract

*Objectives*. In a previous randomized clinical trial (Falsini et al. (2010)), it was shown that short-term Saffron supplementation improves retinal flicker sensitivity in early age-related macular degeneration (AMD). The aim of this study was to evaluate whether the observed functional benefits from Saffron supplementation may extend over a longer follow-up duration. *Design*. Longitudinal, interventional open-label study. *Setting*. Outpatient ophthalmology setting. *Participants*. Twenty-nine early AMD patients (age range: 55–85 years) with a baseline visual acuity >0.3. *Intervention*. Saffron oral supplementation (20 mg/day) over an average period of treatment of 14 (±2) months. *Measurements*. Clinical examination and focal-electroretinogram-(fERG-) derived macular (18°) flicker sensitivity estimate (Falsini et al. (2010)) every three months over a followup of 14 (±2) months. Retinal sensitivity, the reciprocal value of the estimated fERG amplitude threshold, was the main outcome measure. *Results*. After three months of supplementation, mean fERG sensitivity improved by 0.3 log units compared to baseline values (*P* < 0.01), and mean visual acuity improved by two Snellen lines compared to baseline values (0.75 to 0.9, *P* < 0.01). These changes remained stable over the follow-up period. *Conclusion*. These results indicate that in early AMD Saffron supplementation induces macular function improvements from baseline that are extended over a long-term followup.

## 1. Introduction

Age-related macular degeneration (AMD) is a retinal neurodegenerative disease whose distinctive features in the early stage are large soft drusen and hyper/hypopigmentation of the retinal pigment epithelium (RPE), with a moderate loss of central vision (age-related maculopathy, following the International Classification, [1]). In its late stage, the disease is characterized by the geographic atrophy of the RPE or the subretinal neovascular membranes, leading to a more severe central visual impairment which is a major cause of severe, irreversible low-vision in elderly people of developed world.

 Changes of the RPE and photoreceptor cells are early events in AMD and may significantly impact on visual function. Epidemiologic and molecular genetic data indicate that several factors may protect against or increase the individual risk of photoreceptor degeneration/dysfunction in AMD. Many risk factors appear to be oxidative and/or proinflammatory [2–4], while many protective factors are known to act as antioxidants and/or anti-inflammatory [5–7]. Recent clinical studies using focal, psychophysical, or multifocal electroretinograms (ERGs) techniques as assays of the outer retinal function (cone photoreceptors/bipolar cells) have shown that dietary antioxidant supplementation might influence macular cone-mediated function early in the disease process [8–10]. The large-scale, AREDS investigation results [5] indicate that antioxidant supplementation might prevent the development of the most advanced stage of AMD.

 Among various antioxidants, the neuroprotective potential of the ancient spice Saffron was explored [11, 12]. The results showed that Saffron may protect photoreceptors from retinal stress, maintaining both morphology and function and probably acting as a regulator of programmed cell death, in addition to its antioxidant and anti-inflammatory properties [12]. Falsini et al., [13], in a randomized, double-blind, placebo-controlled study showed that three months of dietary Saffron supplementation significantly improved the focal-ERG-(fERG-) estimated retinal flicker sensitivity in early AMD patients. Daily supplementation of 20 mg/d Saffron for 90 days resulted in statistically significant improvements, compared to a placebo control, in the macular fERG parameters (amplitude and modulation threshold) of patients with early AMD. These postsupplementation changes in the macular fERG reflected a beneficial effect of macular function as they were associated with a small, but significant increase in the average Snellen visual acuity. These properties, together with preclinical evidence, provide a strong rationale for testing the effect of prolonged Saffron supplementation in early AMD. The aim of the present longitudinal, open-label study was to evaluate whether the observed functional benefits from Saffron supplementation may be reproducible over a longer follow-up duration.

## 2. Materials and Methods

### 2.1. Patients

 Twenty-nine consecutive patients (mean age,  69.3 ± 7  years; range, 55–85; 16 men and 13 women) with a diagnosis of bilateral early AMD were recruited prospectively over an interval of 8 months from the outpatient service of the institution. Each patient underwent standard general and ophthalmic examinations. Best-corrected visual acuity was determined with a retroilluminated, standardized Snellen chart, whose luminance and contrast were periodically calibrated. Clinical diagnosis of early AMD was established by direct and indirect ophthalmoscopy, as well as retinal biomicroscopy, when any of the following primary lesions in the macular area (i.e., the area within an eccentricity of approximately 2 disc diameters from the fovea) of one or both eyes was identified: soft distinct or indistinct drusen, areas of hyperpigmentation associated with drusen, or areas of hypopigmentation of the RPE associated with drusen, without any visibility of choroidal vessels. Clinical and demographic data of the patients are summarized in Table 1. All patients met the following inclusion criteria: best-corrected visual acuity of 0.5 or better in the study eye, central fixation (assessed by direct ophthalmoscopy), normal color vision with Farnsworth D-15 testing, no signs of other retinal or optic nerve disease and clear optical media. Eight patients had moderate systemic hypertension. No other systemic diseases were present. None of the patients was taking medications (e.g., chloroquine) that are known to affect macular function or to interfere with carotenoid absorption. AMD lesions of the study eyes were graded on stereoscopic fundus photographs, as previously described [14]. A macular grading scale, based on the international classification and grading system [1], was used by a single grader who evaluated the photographs while masked to subject characteristics and fERG results. The presence of basic AMD lesions was noted within each of the nine subfields delimited by a scoring grid. Fluorescein angiography and macular optical coherence tomography (OCT, Cirrus spectral domain, Zeiss) assessment were also performed in all study eyes at the time of the diagnosis, to confirm the presence of early AMD lesions, to exclude geographic atrophy or RPE detachment, and to determine at baseline the average retinal thickness in the macular region. According to the results of grading, intermediate AMD was diagnosed in all eyes, [5] with one or more drusen, (≥63 *μ*m) and/or focal hypo/hyperpigmentation within the macular region. The research adhered to the tenets of the Declaration of Helsinki. The study was approved by the Ethics Committee/Institutional Review Board of the University. Written informed consent was obtained from each study participant after the purpose and procedures of the study were fully explained.

### 2.2. Treatment and Testing Schedule

 The patients underwent clinical examination and a focal-ERG- (fERG-)-derived macular (18°) flicker sensitivity estimate [13] at baseline and every three months over a 15-month period of treatment (Saffron 20 mg/day, Zaffit, Hortus Novus, L'Aquila, Italy) and followup. fERG sensitivity, derived from the estimated response amplitude thresholds, was the main outcome measure. Visual acuity was a secondary outcome measure.

 In all patients, a clinical examination, including Snellen visual acuity testing, fundus examination by direct and indirect ophthalmoscopy, and fERG testing, was performed at study entry (baseline) and every 90 days of treatment. Clinical and fERG examinations were conducted on the same day, with ophthalmoscopy always performed after fERG recordings. During the entire period of supplementation, no other systemic pharmacologic treatments were given. In all cases, compliance was judged to be satisfactory, since none of the treated subjects refrained, for any reason, from taking the daily dose of supplement during the treatment period. No adverse systemic side effects were recorded.

### 2.3. Electrophysiological Methods

 fERG testing was performed according to a previously published technique [13, 14]. Briefly, ERGs were elicited by the LED-generated sinusoidal luminance modulation of a circular uniform field (diameter, 18°; mean luminance, 80 cd/m^2^; dominant wavelength, 630 nm), presented at the frequency of 41 Hz on the rear of a Ganzfeld bowl, illuminated at the same mean luminance as the stimulus. This technique was developed according to the indications of published clinical studies, in which the fERG response to sinusoidal flicker stimulation was used to test retinal flicker sensitivity in comparison to psychophysical flicker sensitivity in normal and pathologic conditions [14–16]. In the recording protocol, a series of fERG responses was collected at different modulation depths, quantified by the Michelson luminance contrast formula: 100%  × (*L*max − *L*min)/(*L*max + *L*min), where *L*max and * L*min are maximum and minimum luminance, respectively, between 16.5% and 93.8% in 0.1- to 0.3-log-unit steps (16.5%, 33.1%, 44.8%, 63.6%, 77.2%, and 93.8%). In some patients, the signal-to-noise ratio (S/N) at the modulation of 93.5% was not large enough to allow recording of the whole response family. In those cases, response collection was limited to the highest or the two highest modulation depths. In Table 1, the number of responses that were significantly different from the noise level, collected at every visit, is reported for each patient.

 fERGs were recorded monocularly by means of Ag-AgCl superficial cup electrodes taped over the skin of the lower eyelid. A similar electrode, placed over the eyelid of the contralateral, patched eye, was used as the reference (interocular ERG, [17]). fERG signals were amplified, bandpass filtered between 1 and 250 Hz (−6 dB/octave), sampled with 12-bit resolution, (2-kHz sampling rate), and averaged. A total of 1600 events (in eight blocks of 200 events each) were averaged for each stimulus condition. The sweep duration was kept equal to the stimulus period. Single sweeps exceeding the threshold voltage (25 *μ*V) were rejected, to minimize noise coming from blinking or eye movements. A discrete Fourier analysis was performed off line to isolate the fERG fundamental harmonic and estimate its amplitude (in *μ*V) and phase (in degrees). Component amplitude and phase were also calculated separately for partial blocks (200-event packets) of the total average, from which the standard error of amplitude and phase estimates were derived to test response reliability. Averaging and Fourier analysis were also performed on signals sampled asynchronously at 1.1 times the temporal frequency of the stimulus, to give an estimate of the background noise at the fundamental component. An additional noise estimate at the fundamental harmonic was obtained by recording responses to a blank, unmodulated field kept at the same mean luminance as the stimulus. In all records, the noise amplitudes recorded with both methods were ≤0.053 *μ*V.

 In all subjects, the fERG testing protocol was started after a 20-minute period of preadaptation to the stimulus mean illuminance. Pupils were pharmacologically (tropicamide 1%) dilated to 8 to 9 mm. Subjects fixated (from a distance of 30 cm) on the center of the stimulation field with the aid of a small (15 minutes of arc) fixation mark. An fERG response was first collected at the maximum modulation depth (93.5%) included in the protocol and was evaluated with respect to reliability and S/N ratio. In all patients, the responses at 93.5% modulation satisfied the following criteria: standard deviation estimates of <20% (variation coefficient) and 15° for the amplitude and phase, respectively, and an S/N ratio ≥4. In AMD patients having a response S/N ≥8, fERG signals were also acquired in sequence for six values of modulation depth between 16.5% and 93.5%, presented in an increasing order. For each stimulus modulation depth, fERG responses were accepted only if their S/N ratio was ≥2. As described elsewhere [14], fERG log amplitudes were plotted for each patient as a function of log modulation depth. The resulting function slope was determined by linear regression. From the same regression line, fERG threshold was estimated from the value of log modulation depth yielding a criterion amplitude, corresponding to an S/N ratio of 3.28. fERG sensitivity was defined as the reciprocal of this value.

### 2.4. Statistical Analysis

 From each patient included in the study, one eye, typically the eye with the best visual acuity, was selected and designated as the study eye. The data from the study eyes were included in the statistical analysis. Outcome variables were fERG amplitude and fERG function threshold and slope. fERG amplitude data underwent logarithmic transformation to better approximate normal distribution. fERG thresholds and slopes are reported as log10 values. In all statistical analyses, standard error and 95% confidence interval (CI) of the means were used for within-group comparisons.

 Sample size estimates were based on those reported in previous investigations [8, 13, 14], in which the between- and within-subjects variability (expressed as the standard deviation) of fERG parameters was determined in patients with early AMD. Assuming between-and within-subject SDs in fERG amplitude and phase of 0.1 log *μ*V and 20°, respectively, the sample size of study patients provided a power of 80%, at an *α* = 0.05, for detecting test-retest differences of 0.1 (SD 0.1) log *μ*V in threshold. Given the absolute mean amplitude and phase values of the patients' fERGs, these differences were considered to be clinically meaningful, since they corresponded approximately to a 25% to 30% change in fERG threshold. A study [8] in patients with early AMD showed that test-retest variability in fERG amplitude and threshold is significantly smaller than this change. Electrophysiological results were analyzed by analysis of variance for repeated measures (ANOVA). Dependent variables in the ANOVA design were fERG log threshold and slope and visual acuity. Repeated-measures ANOVA on log threshold and slope as dependent variables was used to compare the fERG results recorded across the different recording sessions. Visual acuity changes across treatments were analyzed, either individually for every patient or as group means by repeated-measures ANOVA, assuming normal distribution. In all the analyses, results with a *P* < 0.05 were considered as statistically significant.

## 3. Results

**Figure 1:**
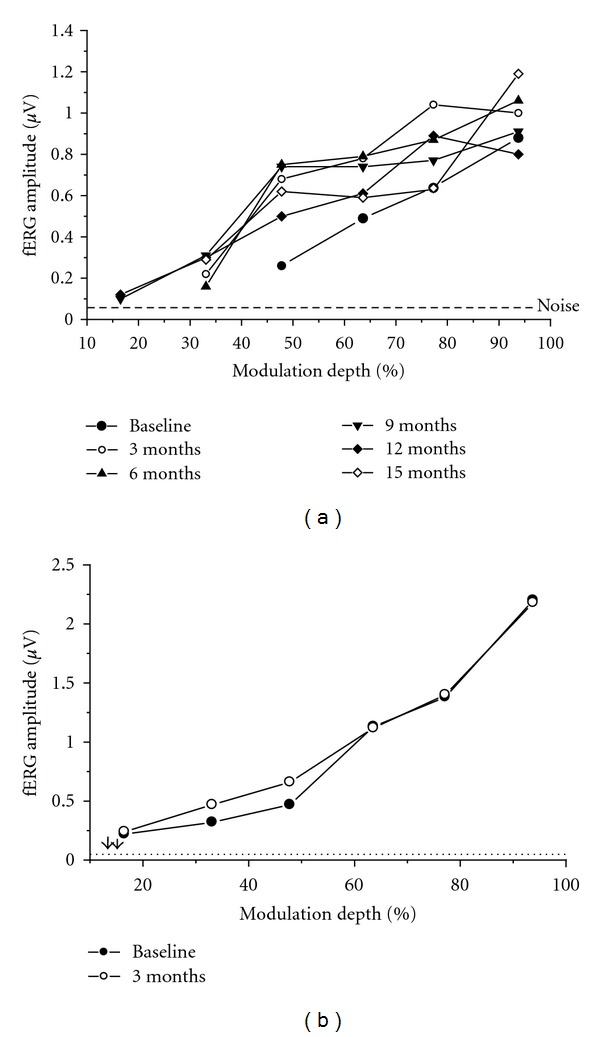
(a) fERG results recorded at baseline and every three months, over a 15 month followup, in an early AMD patient taking saffron supplement (20 mg/day) (b) Plot showing, for comparison, fERG test-retest results obtained from a normal control subject at baseline and after three months.

 Typical fERG functions (response amplitude versus modulation depth of the stimulus) observed in an early AMD patient after Saffron supplementation across the different recording sessions over a fifteen-month followup are reported in Figure 1(a). fERG amplitude increased from baseline after three months of supplementation, resulting in a reduction of response threshold, as shown by the decrease of the minimum modulation depth yielding a response significantly above the noise level (S/N ratio > 3).  Figure 1(b) shows, for comparison, the fERG test-retest results obtained from a normal control subject at baseline and after three months. It can be noted that the test-retest variability of fERG data was considerably smaller than that observed after Saffron supplementation in the AMD patient.

**Figure 2:**
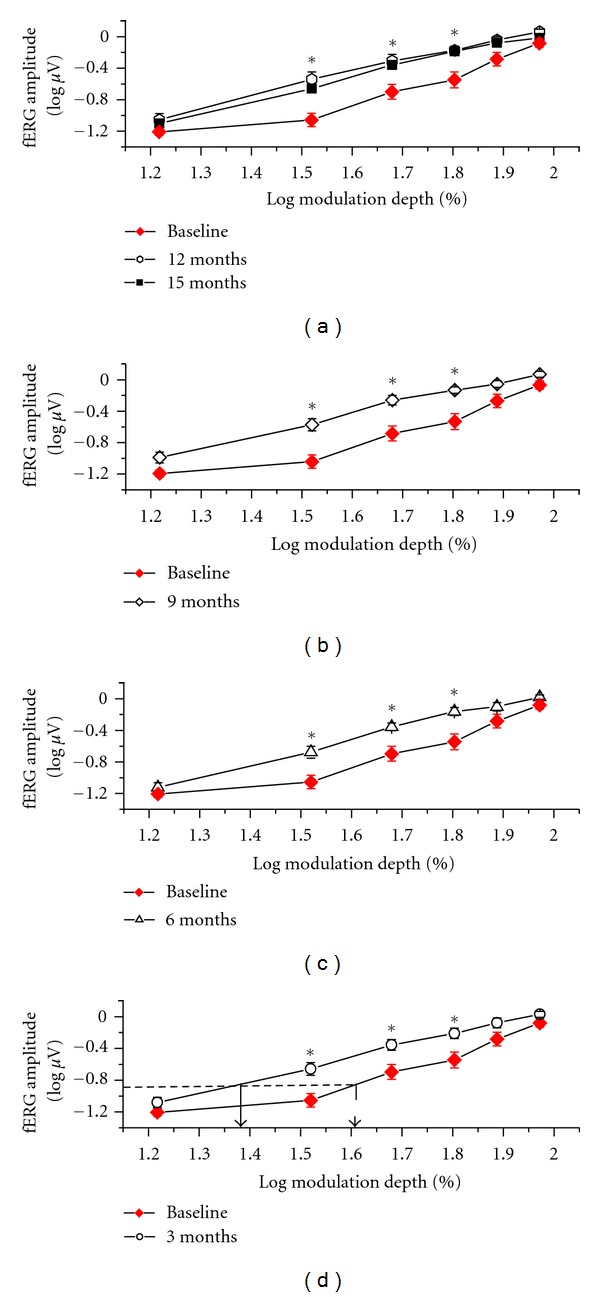
Mean (±SEM) fERG functions, plotting log amplitude versus log modulation depth of the flicker stimulus, recorded from all 29 patients at baseline and at the various time points of the study. Note that mean fERG function was shifted uniformly to the left on the *x*-axis after the first three months of supplementation and then remained stable. Arrows in the plot indicate the mean shift in fERG sensitivity observed by comparing the baseline with the follow-up fERG recordings (averaged across the different times). Asterisks indicate data points that were significantly (*P* < 0.05) different from baseline.

Figure 2 shows the mean fERG functions (±SEM) recorded at baseline and every three months after starting Saffron supplementation. There was an overall increase in fERG amplitude soon after the first three months of supplementation followed by a stabilisation over the subsequent follow-up period. The average fERG functions were uniformly and consistently shifted to the left on the *x*-axis indicating a decrease in response threshold and an increase in sensitivity, which were reproducible in the various recording sessions. The arrows in the plot highlight the change in the average modulation threshold comparing the baseline with the follow-up recordings (averaged across the different time points).

**Figure 3:**
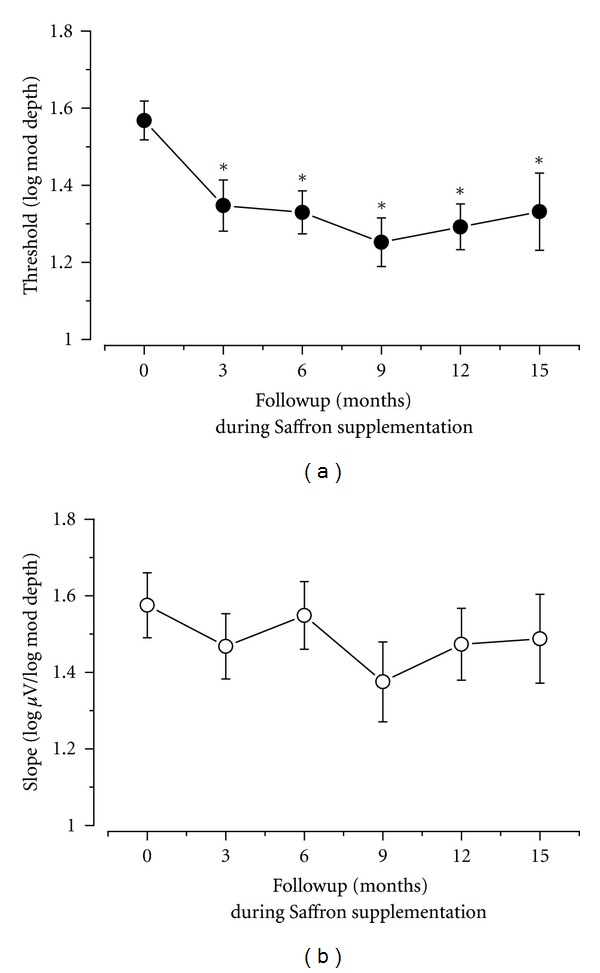
Mean fERG thresholds and slopes (± standard error) recorded at baseline and over the follow-up period in all patients. Note that mean decreased (i.e., sensitivity increased) from baseline already after three months of supplementation and then tended to stabilize. Mean fERG slope did not change significantly over time.

This point is illustrated in further detail in Figure 3, which shows the fERG threshold and slope results (mean ± SEM) recorded at every follow-up session over the period of follow-up examination. Mean fERG threshold decreased (and consequently the reciprocal sensitivity value increased) by 0.3 log units compared to the baseline value (repeated measures ANOVA, *F* = 4.6;  *df*: 6,168; *P* < 0.01). These changes remained stable over the followup period, since comparisons at various times of follow-up did not show any significant change. The mean fERG slopes did not change significantly throughout the followup.

**Figure 4:**
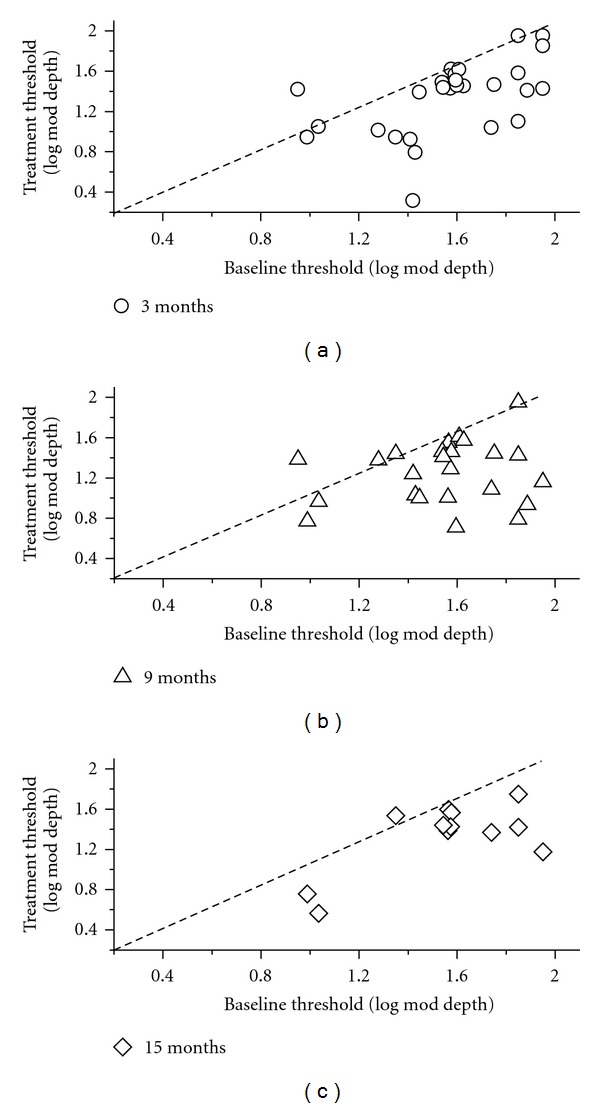
Scatterplots showing the fERG threshold values recorded at different follow-up times (3, 9, and 15 months) plotted as a function of the corresponding baseline values. Diagonal lines in the plots indicate the equivalence between the values recorded at baseline and at a given followup. It can be noted that, at every followup, most values fall on the right of the diagonal line, indicating a decrease in threshold for the majority of patients.

In most patients at the various times after starting supplementation, fERG thresholds decreased by a variable amount compared to the corresponding baseline value.  Figure 4 provides examples of the observed changes in response thresholds observed at 3, 9, and 15 months. The results found at 6 and 12 months are substantially similar. In the scatterplots of the figure, the values recorded at the different follow-up times are plotted as a function of the corresponding baseline values. Diagonal lines in the plots indicate the equivalence between the values recorded at baseline and at a given followup. It can be noted that, at every followup, most values fall on the right of the diagonal line, indicating a decrease in threshold for the majority of patients.

**Figure 5:**
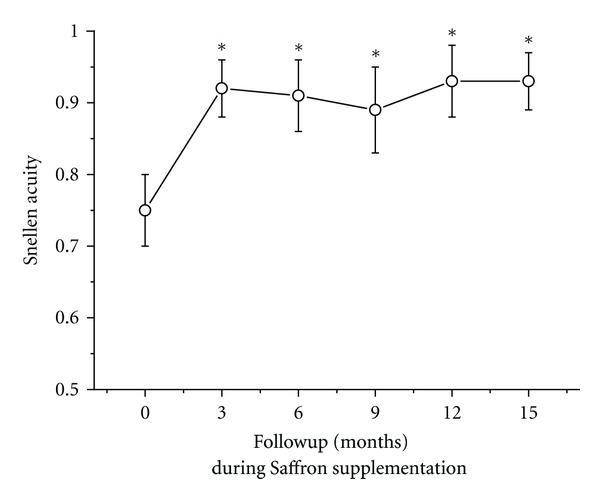
Mean (± standard error) Snellen visual acuity recorded at baseline and every three months throughout the follow-up period.

Right after three months of supplementation, mean visual acuity improved by two Snellen lines compared to baseline values (0.75 to 0.9, repeated measures ANOVA, *F* = 4.3; *df*: 6,168; *P* < 0.01). The visual acuity results over the follow-up period are reported in Figure 5 as a plot of mean (± SEM) acuity as a function of follow-up time. It can be noted that, similar to fERG sensitivity, the gain in visual acuity remained stable over the study period.

In parallel with the electrophysiological and visual acuity improvement, all patients reported an improvement in their quality of vision. The most commonly reported symptoms of the beneficial effects of supplementation were the improvement in contrast and color perception, reading ability, and vision at low luminances, all ultimately leading to a substantial improvement in the patients' quality of life.

**Figure 6:**
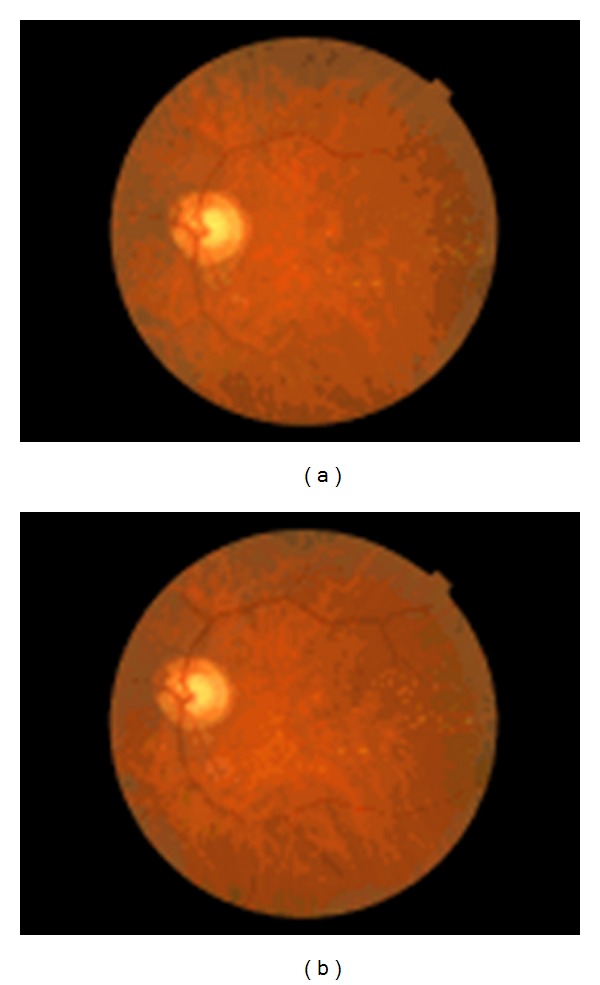
Fundus pictures of a representative early AMD patient taken at baseline and at the end of follow-up. No significant changes in fundus features can be found.

Funduscopic examination, periodically performed in all patients, did not show any significant change in drusen number and size, as well as in the extension of RPE abnormalities.  Figure 6 shows fundus pictures of one study patient taken at baseline and at the end of clinical followup. It can be seen that both funduscopic images appeared to be very similar to each other.

## 4. Discussion

 The present data extend the results of a previous, randomized study [13] on the potential efficacy of Saffron supplementation in AMD, showing that such supplementation may induce a long-term, stable improvement in retinal function, as measured by fERG sensitivity. To our knowledge, this is the first longitudinal study, based on an objective electrophysiological technique, documenting the long-term effects of Saffron antioxidant on retinal function in AMD. While several other studies have already shown significant effects of antioxidant supplementation on retinal function [8, 10, 13], all these previous studies were more limited either in their follow-up duration or in the number of testing sessions. The current data show that changes in fERG threshold (and consequently its reciprocal value sensitivity) after supplementation were within-session consistent, reproducible, and durable over a 15-month follow-up period. The sensitivity improvements were observed in association with a constant slope of the fERG functions, further indicating the reproducibility of the fERG function shapes recorded during followup. All patients reported a subjective improvement in their quality of vision and ultimately in their quality of life, supporting the impact of the fERG findings on psychophysical visual function of patients.

 Limitations of this study include its open label nature, which may affect mainly the subjective patients' results, and the assumed stability of the main outcome measure, the fERG, without treatment. As far as the first issue is concerned, the efficacy of Saffron supplementation was already demonstrated in a previous double-blind, randomized, placebo-controlled study [13], strongly supporting the hypothesis that the long-term fERG improvement found in our patients similarly results from Saffron supplementation, and not from other unknown and unpredictable factors. Regarding the second issue, previous studies of our group [8, 14], performed in normal subjects and early AMD patients without treatment, have shown an fERG test-retest variability that is small enough to allow macular function to be reliably monitored during treatment. Taking into account the previous limitations, the current findings point at the use of macular fERG sensitivity as a candidate protocol to track changes in central retinal function over the course of AMD.

 It may be presumed that the observed improvement in macular function is an effect of integrated activities of Saffron's chemical compounds, mainly of crocin, and crocetin, antioxidant derivatives of carotenoids, which may act through a protective mechanism similar to that seen with carotenoid supplementation [18–21], resulting in a beneficial effect on retinal function. In addition, crocins are able to activate metabolic pathways to protect cells from apoptosis and to reduce light-induced death in isolated photoreceptors, while crocetin increases oxygen diffusivity through liquids, such as plasma [18].

 In the original study by Maccarone et al. [11], to test whether the Saffron extract (Crocus sativus L.) given as a dietary supplement counteracts the effects of continuous light exposure in the albino rat retina, Sprague-Dawley rats were prefed either Saffron or beta-carotene before they were exposed to bright continuous light for 24 hours. Flash electroretinograms (ERGs) amplitudes, the thickness of the outer nuclear layer (ONL), and the amount of apoptotic figures in the ONL were the main outcome variables. The photoreceptor layer was largely preserved in Saffron-treated animals as it was the flash ERG response. In addition, the rate of photoreceptor death induced by bright continuous light appeared drastically reduced in treated animals. In beta-carotene prefeeding experiments, morphologic analysis showed preservation of the ONL similar to that obtained with Saffron prefeeding, whereas the ERG response was unrecordable. Western blot analysis showed that exposure to light induced a strong upregulation of fibroblastic growth factor (FGF2) in control and beta-carotene-treated rats, but no change was noted in Saffron-treated rats. These results showed that Saffron may protect photoreceptors against retinal stress, maintaining both morphology and function and probably acting as a regulator of programmed cell death. To identify the genes and noncoding RNAs (ncRNAs) involved in the neuroprotective actions of Saffron, Natoli et al. [12] used continuous bright light as a standardized assay of photoreceptor damage in albino Sprague Dawley rats. RNA from the eye of exposed and unexposed animals was hybridized to Affymetrix rat genome ST arrays. Light damage caused the regulation of 175 entities (genes and ncRNAs) beyond criterion levels. Saffron treatment before light damage exposure reduced the expression of 53 entities and regulated 122 entities not regulated by light damage. This analysis provides a basis for more focused basic studies on Saffron protective mechanism(s).

 The peculiar characteristics of Saffron components support our hypothesis of an involvement of very different ways of action going from antioxidant activity to direct control of gene expression. It should be noted that the current results can only be applied to the early/moderate stage of AMD. It is currently unknown whether Saffron supplementation may exert a beneficial protective effect in patients with more advanced stages of disease. In addition, it is unclear how the Saffron efficacy compares with that of other antioxidant supplements currently available, such as the AREDS preparation [5]. While further studies are needed to define the upper beneficial limit of Saffron supplementation, the present approach seems to be promising for a long-term treatment of early retinal dysfunction associated with AMD.

## Figures and Tables

**Table 1:** Demographic and clinical findings at baseline in patients with early AMD.

Patient no.	Age (yr), sex	Acuity	Follow-up duration	Fundus^∗^	Macular thickness	fERG^§^
					(microm)	(no. of responses at B, 3, 6, 9, 12, 15)^¶^
1	77, F	0.8	15	Soft drusen, middle subfield	304	4(B), 5(3), 5(6), 5(9), 5(12), 5(15)
2	62, F	0.6	15	Soft drusen, middle subfield	272	6(B), 6(3), 6(6), 6(9), 6(12), 6(15)
3	61, F	0.5	12	Soft drusen, middle subfield	250	5(B), 6(3), 6(6), 6(9), 6(12)
4	63, F	0.7	12	Soft drusen, central and middle subfield	288	5(B), 6(3), 6(6), 6(9), 5(12)
5	75, M	0.7	15	Soft drusen, middle subfield	279	4(B), 5(3), 5(6), 6(9), 6(12), 6(15)
6	85, M	0.8	12	Soft drusen, central and middle subfield	260	5(B), 5(3), 5(6), 5(9), 5(12), 5(15)
7	70, M	0.7	15	Soft drusen, central subfield	280	4(B), 4(3), 4(6), 5(9), 5(12), 5(15)
8	71, M	1.0	12	Soft confluent drusen, middle subfield	254	5(B), 6(3), 6(6), 5(9), 5(12)
9	73, M	1.0	15	Soft drusen, central subfield	294	4(B), 5(3), 5(6), 6(9), 6(12), 6(15)
10	81, F	0.5	6	Soft drusen, middle subfield	251	4(B), 4(3), 4(6)
11	73, M	0.7	15	Soft drusen, central and middle subfield	275	1(B), 5(3), 6(6), 6(9), 6(12)
12	62, F	0.6	15	Soft drusen, middle subfield	297	6(B), 6(3), 6(6), 6(9), 6(12), 6(15)
13	73, M	1.0	15	Soft drusen, hyperpigm., middle subfield	221	4(B), 4(3), 5(6), 5(9), 5(12), 4(15)
14	68, M	0.8	15	Soft confluent drusen, central and middle subfield	242	2(B), 4(3), 4(6), 6(9), 6(12), 5(15)
15	58, M	1.0	6	Soft drusen, middle subfield	280	1(B), 5(3), 5(6)
16	63, M	0.8	15	Soft confluent drusen, hypopigm., middle subfield	278	4(B), 5(3), 5(6), 4(9), 5(12), 5(15)
17	64, F	1.0	15	Soft drusen and hypopigm., middle subfield	264	2(B), 6(3), 6(6), 5(9), 6(12), 6(15)
18	55, M	1.0	15	Soft drusen and hyperpigm., central subfield	295	5(B), 5(3), 6(6), 6(9), 6(12), 5(15)
19	70, F	0.7	15	Soft drusen and hyperpigm., middle subfield	237	2(B), 2(3), 1(6), 3(9), 3(12), 4(15)
20	79, M	0.4	15	Soft drusen and hyperpigm., middle subfield	255	1(B), 1(3), 4(6), 6(9), 6(12), 6(15)
21	70, M	1.0	12	Soft drusen and hyperpigm., central subfield	279	4(B), 5(3), 5(6), 4(9), 4(12)
22	70, M	0.7	15	Soft confluent drusen, central subfield	290	5(B), 5(3), 5(6), 5(9), 5(12), 5(15)
23	85, M	0.3	12	Soft drusen, middle subfield	255	1(B), 2(3), 2(6)
24	71, F	1.0	15	Soft drusen, central and middle subfield	280	4(B), 5(3), 5(6), 6(9), 5(12), 5(15)
25	73, F	1.0	15	Soft drusen and hyperpigm., middle subfield	266	3(B), 5(3), 5(6), 5(9), 5(12), 5(15)
26	71, F	0.6	15	Soft confluent drusen, hypopigm., central subfield	270	6(B), 6(3), 5(6), 5(9), 6(12), 5(15)
27	61, M	0.5	15	Soft confluent drusen, hypopigm., middle subfield	265	2(B), 6(3), 5(6), 6(9), 6(12), 5(15)
28	68, F	0.6	15	Soft confluent drusen., central subfield	293	6(B), 5(3), 5(6), 5(9), 5(12), 5(15)
29	56, F	0.6	12	Soft confluent drusen, middle subfield	277	6(B), 6(3), 6(6), 5(9), 6(12)

^∗^Macular appearance with reference to drusen type, confluence, and location; RPE abnormalities type and main location^1^. Follow-up duration (months). ^§^Number of FERG responses that were above noise level (i.e., S/N ratio ≥ 3) at the different modulation depths of the recording protocol; (6) = S/N ratio ≥ 3 at all modulation depths, (5) = S/N ratio < 3 at the lowest modulation depth, (4) = S/N ratio < 3 at the two lowest modulation depths, etc., B: baseline 3, 6, 9, 12, 15 months of supplementation. ^¶^Months of follow-up.
